# Predictors and health impacts of Ramadan intermittent fasting among patients with sickle cell disease

**DOI:** 10.3389/fmed.2025.1584795

**Published:** 2025-05-29

**Authors:** Mortadah Hadi Alsalman, Zaenb Alsalman, Maryam Mohammed Alshams, Jinan Mohammed Aljasem, Zahra Saleh Alsindi, Amani Abdullah Almutairi, Khadija Abdulrahman Alsunaikh, Hadeel Hesham Buali, Kefah Baqir Algadeeb, Nawal Eltayeb Omer Mohammed, Abdulaziz Abdullah Bushehab, Marwa Mahmoud Shafey

**Affiliations:** ^1^Department of Medicine, College of Medicine, King Faisal University, Al Ahsa, Saudi Arabia; ^2^Department of Family and Community Medicine, College of Medicine, King Faisal University, Al Ahsa, Saudi Arabia; ^3^Department of Medicine, King Fahad Hospital, Al Ahsa, Saudi Arabia; ^4^Department of Medicine, Hereditary Blood Diseases Center, Al Ahsa, Saudi Arabia; ^5^Department of Family and Community Medicine, College of Medicine, Imam Abdulrahman bin Faisal University, Dammam, Saudi Arabia

**Keywords:** sickle cell disease, intermittent fasting, vaso-occlusive crisis, Ramadan, fasting recommendations

## Abstract

**Introduction:**

Ramadan fasting constitutes a form of intermittent fasting, and it is a religious obligation for healthy adult Muslims; however, there is a lack of comprehensive guidance for individuals diagnosed with sickle cell disease (SCD). This study seeks to identify predictors of fasting capability and investigate the effects of fasting among this patient population.

**Participants and methods:**

This cross-sectional study enrolled adult Muslim patients with SCD from Saudi Arabia. Data were collected through direct interviews using a structured questionnaire with four sections.

**Results:**

A total of 320 patients with SCD were evaluated, with a mean age of 32.6 years (SD ± 11.01) and the majority were females (63.1%). Among these participants, 214 (66.9%) observed Ramadan intermittent fasting (RIF) while 106 (33.1%) did not observe the fasting month or had interrupted fasting. Participants having secondary and university education were nearly twice as likely to fast the entire Ramadan (*p* = 0.025 and *p* = 0.003, respectively). History of SCD crises, emergency visits, acute chest syndrome, and intensive care unit admission were all significantly associated with lower odds of fasting the entire month (OR = 0.25, *p* = 0.019; OR = 0.25, *p* = 0.001; OR = 0.57, *p* = 0.035; and OR = 0.46, *p* = 0.003, respectively). Participants with more than four SCD episodes or more than four emergency room visits had significantly higher odds of experiencing complications within 30 days following Ramadan (OR = 3.31, *p* < 0.001 and OR = 2.55, *p* = 0.004, respectively).

**Conclusion:**

The effects of observing RIF on individuals with SCD are generally subtle during the month but more significant in the post-fasting phase. The capacity to predict an individual’s ability to fast and likelihood of post-fasting complications is substantially influenced by a range of sociodemographic factors such as education level as well as clinical variables, including frequency of annual hospitalizations, emergency room visits, intensive care unit admissions, occurrences of acute chest syndrome, and the presence of thrombocytosis. A Comprehensive evaluation of these factors will provide enhanced guidance for medical practitioners, facilitating more informed and insightful decisions regarding patient fasting recommendations.

## Introduction

Sickle cell disease (SCD) is considered the most frequent genetic disease throughout the world. It is a chronic blood disorder that significantly impacts patients’ lives across clinical, economic, psychological, and psychosocial dimensions (1–4). This inherited hemoglobinopathy results from a single mutation in the *β*-globin gene, which leads to the production of abnormal hemoglobin S (HbS). Significant changes in the properties and morphology of red blood cells result as a consequence of HbS deoxygenation and subsequent polymerization of hemoglobin molecules (5, 6). Disease phenotype varies greatly between SCD genotypes, and is characterized by a myriad of acute and chronic events ranging from painful vaso-occlusive crises and acute chest syndrome (ACS) to chronic complications including organ failure, life-threatening infections, stroke, and a limited life expectancy (5, 7).

Each year, there are about 300,000 new cases of SCD globally. In Saudi Arabia, approximately 4.2% of the population possesses the sickle-cell trait, while 0.26% has SCD. The Eastern province exhibits the highest prevalence, with roughly 17% of the population carrying the gene and 1.2% affected by SCD (8, 9).

Muslims account for almost a quarter of the world’s population, with an estimated 2 billion Muslims (10). In Islam, Ramadan is the holy month of fasting, during which all healthy adult Muslims are obligated to fast every day from dawn to sunset, refraining from smoking, food, drink, and oral medication (11). Fasting duration varies from year to year, ranging from 10 to 21 h a day depending on geographical location and solar seasons, and the lunar month of Ramadan coincides with. However, there are several exemptions to fasting this month, including children, pregnant and breastfeeding women, during menstruation, travelers, and people with acute or chronic conditions for whom fasting would be risky. Additionally, Muslims are allowed to break their fast if they feel ill, and they can resume their fast once they have fully recovered (12–14).

Ramadan intermittent fasting (RIF) is considered a unique model of intermittent daytime fasting. In humans, the three most widely studied intermittent fasting (IF) regimens are alternate-day fasting, 5:2 IF (fasting 2 days each week), and daily time-restricted eating, such as the 16/8 approach (15). RIF is compatible with the last, as it involves fasting for 16 h per day for a complete month (29–30 days) (16). However, RIF might be more difficult than other kinds of IF, as it demands abstaining from all fluids (intermittent dry fasting), and the majority of the fasting occurs during the waking hours (11). Beyond its spiritual benefits, RIF has been shown through systematic reviews and meta-analyses to produce notable metabolic and physiological effects. These include improvements in glucose regulation, body weight and composition, and liver function. Moreover, RIF has been linked to reductions in key inflammatory and oxidative stress markers, contributing to better cardiometabolic health (17–21). Several guidelines and recommendations have been developed to improve chronic illness awareness, knowledge, and management during Ramadan, such as for diabetes mellitus, renal disease and cardiac disease (11, 12, 22, 23). Evidence regarding fasting practices among adult patients with SCD remains limited in both local and global contexts. Consequently, established protocols and directives are lacking, and clinicians are unable to provide definitive guidance concerning fasting to SCD patients. To date, only one study has specifically investigated the impact of RIF on individuals with SCD (24). However, this study was limited in scope as it did not examine the predictors that influence whether patients choose to observe RIF. Therefore, the primary objective of this study is to explore the effects of RIF on SCD patients and to identify the factors that influence an individual’s ability to fast. The findings of this research are anticipated to enhance management strategies and reduce risks associated with fasting periods for these patients.

## Methodology

### Study population

A cross-sectional study was conducted on SCD patients at the Blood Disease Center in Alahsa, Saudi Arabia, from October 2022 to October 2023. The included participants comprised Saudi Muslim SCD patients aged 18 to 65 years. The exclusion criteria included pregnancy for women, having the sickle cell trait, a history of chronic kidney disease, or a history of diabetes. The target sample size was calculated using the Cochrane equation for a finite population (*N* = 2,313) with a margin error of 5% and a confidence interval of 95%, which yielded an estimate of 330 participants. The study was approved by the Ethical Committee of King Fahad Hospital (H-05-HS-065). Participants were informed of the purpose of the study and the confidentiality of the data before giving their consent.

### Data collection

Data were collected by trained research members through direct interviews with patients using a structured questionnaire with four sections. The first section covered sociodemographic data such as age, sex, marital status, education level, and history of chronic disease. The second section incorporated SCD baseline characteristics including the annual frequency of crises, annual frequency of emergency room (ER) visits, previous history of acute chest syndrome, previous history of intensive care unit (ICU) admission, previous history and frequency of blood transfusions, history of hydroxyurea administration (duration and dose), and history of surgical intervention related to SCD complications such as cholecystectomy, hip replacement surgery and splenectomy. The third section covered Ramadan-related data such as fasting status (fasting the entire month (30 days), not fasting at all, or interrupted fasting due to SCD complications) and SCD characteristics during Ramadan (hydroxyurea use, hospital admission, ICU admission, and blood transfusion). Finally, the fourth section concerned post-Ramadan data, specifically SCD characteristics within 30 days after Ramadan (SCD crises, hospital admission, ICU admission, ER visits, and blood transfusion).

### Statistical analysis

Collected data were analyzed using SPSS version 27. Categorical data were presented as numbers and percentages while continuous data were summarized using the mean, median, range, and standard deviation (SD). After performing normality tests, differences in SCD characteristic distribution according to RIF pattern were assessed using the chi-square test for categorical variables and the Kruskal-Wallis test for continuous variables.

Predictors of successful fasting were analyzed using binary logistic regression. The primary outcome was defined as fasting the entire Ramadan month (1) versus not fasting the entire Ramadan month (0). Predictors of complications within 30 days after Ramadan among SCD participants who fasted the entire month were likewise analyzed by binary logistic regression, with the primary outcome being the occurrence of any SCD-related complication (SCD crisis, ER visit, hospital admission, ICU admission, or blood transfusion) within 30 days after Ramadan. The results were reported as odds ratios (ORs) with 95% confidence intervals (CIs). A *p*-value <0.05 was considered statically significant.

## Results

### Participant characteristics

The study included 320 patients with SCD with an age range of 18 to 76 years and mean age of 32.6 years (SD 11.01). The majority were females (63.1%) and were married (52.8%). Regarding baseline SCD complications, almost all participants (91.6%) reported the occurrence of yearly SCD crises (range 1 to 50), with a median of four crises/year and mean ± SD of 6.70 ± 7.43 crises. Also, most participants (83.1%) reported annual emergency visits (range 1 to 60), with a median value of four visits/year and a mean of 7.19 ± 8.36 visits/year. Two-thirds of participants (66.6%) reported a history of ACS, and more than half (58.1%) reported a history of ICU admission. In addition, the great majority of participants (80.6%) reported a history of blood transfusion during their lives, with a wide range of 1 to 120 transfusions, an average of 9.66 ± 16.02, and a median of four. Regarding hydroxyurea use, more than a third of participants (*n* = 118, 36.9%) were currently taking it; however, only about half of them (50.8%, *n* = 60) were on the optimal dose. The average hydroxyurea dosage was 15.80 ± 4.70 mg/kg (median 14.52), with a range of 2.23–28.57 mg/kg. Notably, only 35.6% of participants reported using hydroxyurea during Ramadan. All collected demographic and health characteristic data are summarized in Table 1.

**Table 1:** Demographic and health characteristics of the participants according to Ramadan fasting pattern.

Characteristic	Total (*n* = 320) *n* (%)	Fasting all Ramadan month (*n* = 214) *n* (%)	Not fasting at all (*n* = 20) *n* (%)	Interrupted fasting (*n* = 86) *n* (%)	*p* value
Age in years (Mean±SD)^&^	32.62 ± 11.01	32.17 ± 11.01	35.3 ± 13.09	33.18 ± 10.22	0.523
Sex					
Male	118 (36.9)	78 (66.1)	3 (2.5)	37 (31.4)	0.063
Female	202 (63.1)	136 (67.3)	17 (8.4)	49 (24.3)	
Marital status					
Single	151 (47.2)	108 (71.5)	10 (6.6)	33 (21.9)	0.160
Married	169 (52.8)	110 (62.7)	10 (5.9)	53 (31.4)	
Education level					
Lower than secondary	72 (22.4)	38 (52.8)	9 (12.5)	25 (34.7)	0.020*
Secondary	124 (38.8)	85 (68.5)	5 (4.0)	34 (27.4)	
University and higher	124 (38.8)	91 (73.4)	6 (4.8)	27 (21.8)	
Chronic diseases					
Yes	69 (21.6)	41 (59.4)	8 (11.6)	20 (29.0)	0.086
No	251 (78.4)	173 (68.9)	12 (4.8)	66 (26.3)	
History of SCD crises					
Yes	293 (91.6)	190 (64.9)	18 (6.1)	85 (29.0)	0.017*
No	27 (8.4)	24 (88.9)	2 (7.4)	1 (3.7)	
Average annual SCD crises^&^	6.70 ± 7.43 (*n* = 293)	5.86 ± 6.28 (4) (*n* = 190)	6.11 ± 3.39 (5.5) (*n* = 18)	8.64 ± 9.67 (6) (*n* = 85)	<0.001^*^
History of emergency visits					
Yes	266 (83.1)	167 (62.8)	16 (6.0)	83 (31.2)	0.001^*^
No	54 (16.9)	47 (87.0)	4 (7.4)	3 (5.6)	
Average annual ER visits^&^	7.19 ± 8.36 (*n* = 266)	5.92 ± 6.69 (4) (*n* = 167)	8.50 ± 8.84 (6) (*n* = 16)	9.53 ± 10.54 (6) (*n* = 83)	<0.001^*^
Previous history of acute chest syndrome					
Yes	213 (66.6)	134 (64.9)	14 (6.6)	65 (30.5)	0.093
No	107 (33.4)	80 (74.8)	6 (5.6)	21 (19.6)	
Previous history of ICU admission					
Yes	186 (58.1)	112 (60.2)	15 (8.1)	59 (31.7)	0.010^*^
No	134 (41.9)	102 (76.1)	5 (3.7)	27 (20.1)	
Previous history of blood transfusion					
Yes	258 (80.6)	169 (65.5)	17 (6.6)	72 (27.9)	0.564
No	62 (19.4)	45 (72.6)	3 (4.8)	14 (22.6)	
Average no. blood transfusion^&^	9.66 ± 16.02 (*n* = 258)	8.45 ± 14.89 (3) (*n* = 169)	14.18 ± 15.61 (10) (*n* = 17)	11.42 ± 18.35 (3) (*n* = 72)	0.076
Previous history of surgery					
Yes	203 (63.4)	139 (68.5)	13 (6.4)	51 (25.1)	0.648
No	117 (36.6)	75 (64.1)	7 (6.0)	35 (29.9)	
Current use of hydroxyurea					
Yes	118 (36.9)	82 (96.5)	9 (7.6)	27 (22.9)	0.393
No	202 (63.1)	132 (65.3)	11 (5.4)	59 (29.2)	
Using of hydroxyurea during Ramadan					
Yes	114 (35.6)	79 (69.3)	9 (7.9)	26 (22.8)	0.366
No	206 (64.4)	135 (65.6)	11 (5.3)	60 (29.1)	
Blood parameters (Mean± SD)^&^					
WBC	8.21 ± 4.38	7.94 ± 4.15	9.75 ± 4.03	8.50 ± 4.92	0.081
Hemoglobin	9.84 ± 1.43	9.92 ± 1.39	9.42 ± 1.72	9.72 ± 1.47	0.086
Platelet	298.38 ± 185.89	293.07 ± 169.34	374.50 ± 144.95	293.88 ± 227.32	0.015*
MCV	78.06 ± 11.29	77.77 ± 11.39	81.64 ± 12.65	77.82 ± 10.58	0.340
MCH	27.20 ± 4.71	27.10 ± 4.80	28.85 ± 4.99	27.28 ± 4.36	0.208
Hb A	3.9713 ± 12.08	3.48 ± 11.67	8.77 ± 16.24	4.10 ± 11.87	0.197
Hb A2	3.28 ± 1.80	3.20 ± 1.08	4.91 ± 5.87	3.10 ± 0.88	0.585
Hb F	15.98 ± 7.40	16.20 ± 7.44	14.57 ± 7.71	15.75 ± 7.25	0.720
Hb S	76.66 ± 11.51	76.88 ± 11.52	72.65 ± 13.47	77.03 ± 10.95	0.498

^&^Test of Significance performed is the Kruskal Wallis (K-W) test.

WBC, white blood cells; MCV, mean corpuscular volume; MCH, mean corpuscular hemoglobin; Hb A, hemoglobin A; Hb A2, hemoglobin A2; Hb F, hemoglobin F; and Hb S, hemoglobin S. *p < 0.05 (significant).

### Ramadan fasting pattern

**Figure 1:**
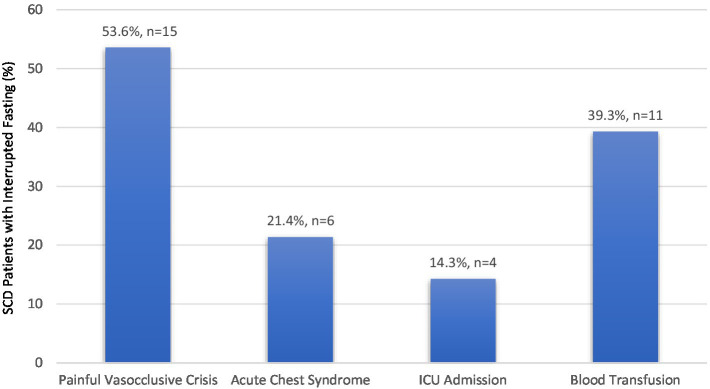
Reasons for hospital admission during Ramadan among SCD patients with interrupted fasting (*n* = 28).

Participants were classified into three groups based on RIF pattern: fasting every day (*n* = 214, 66.9%), not fasting at all (*n* = 20, 6.3%), or interrupted fasting (*n* = 86, 26.9%). On average, participants in the interrupted group broke fasting on 7.06 ± 6.50 days, with a median of 5 days (range 1–29 days) (Table 1). Interruptions were due to SCD complications, of which 32.6% (*n* = 28) required hospitalization, primarily for a painful SCD crisis, followed by blood transfusion (Figure 1). The remaining 67.4% (*n* = 58) of participants in the interrupted group experienced complications that did not require hospitalization; of these, 55.2% broke their fast based on personal preference, while only 10.3% did so following physician recommendation.

Comparative statistical tests revealed significant differences in RIF pattern in relation to participant level of education, history of SCD crises, history of ER visits, and history of ICU admission. Among participants with university-level education, those who did not report any SCD crises, and those who reported no ER visits, a higher proportion successfully fasted the entire month (*p* = 0.020, *p* = 0.017, and *p* = 0.001, respectively). Conversely, participants with interrupted fasting had significantly more SCD crises, a higher mean number of ER visits, and higher ICU admissions (*p* < 0.001, *p* < 0.001, and *p* = 0.010, respectively). Meanwhile, there was no statistically significant difference between the three groups in terms of mean age, sex distribution, marital status, chronic diseases, ACS history, blood transfusion history, or use of hydroxyurea (either current or during Ramadan). In addition, blood parameters did not exhibit statistically significant differences in relation to fasting pattern, except for the mean platelet count, where higher platelet count was observed among non-fasting SCD patients (*p* = 0.015) (Table 1).

Table 2 presents the binary logistic regression results for predictors of fasting the entire month of Ramadan (Table 2). Participants having completed secondary education were nearly twice as likely to fast the entire month, and those with a university education 2.538 times more likely, both of which findings are statistically significant (*p* = 0.025 and *p* = 0.003, respectively).

**Table 2:** Predictors of fasting capability among participants with SCD.

Variables	*p* value	Odds ratio	95% CI
Age	0.281	0.99	0.97–1.01
Sex (Ref: Female)	0.822	0.95	0.58–1.53
Marital (Ref: Single)	0.173	0.72	0.42–1.07
Education (Ref: Lower than secondary)			
Secondary	0.025	1.99	1.07–3.55
University	0.003	2.54	1.34–4.54
Chronic diseases	0.070	0.60	0.38–1.14
History of SCD Crises	0.019	0.25	0.07–0.75
No. of SCD episodes (*n* = 293)	0.017	0.96	0.93–0.99
Number of SCD episodes (*n* = 293) Ref. median or less ≤ 4	0.001	0.45	0.28–0.74
History of emergency visits	0.001	0.25	0.11–0.58
No. ER visits (*n* = 266)	0.003	0.95	0.92–0.98
Number of ER visits (*n* = 266) Ref. median or less ≤ 4	0.002	0.45	0.27–0.75
Previous history of acute chest syndrome	0.035	0.57	0.34–0.96
Previous history of ICU Admission	0.003	0.46	0.29–0.78
Previous history of blood transfusion	0.289	0.72	0.39–1.33
Average no. blood transfusion (*n* = 258)	0.105	0.99	0.97–1.00
Previous history of surgery	0.443	1.21	0.75–1.96
Current use of hydroxyurea	0.576	1.15	0.74–1.97
Optimal dose of hydroxyurea (*n* = 118)	0.781	0.89	0.41–1.96
The use of hydroxyurea during Ramadan	0.619	1.13	0.73–1.94
WBC	0.154	0.10	0.91–1.01
Hemoglobin	0.271	1.69	0.95–1.34
Platelets	0.678	1.00	0.10–1.00
HBA	0.269	0.99	0.98–1.01
HBA2	0.262	0.92	0.81–1.07
HBF	0.605	1.01	0.98–1.04
HBS	0.450	1.01	0.98–1.02

On the other hand, history of SCD crises, emergency visits, acute chest syndrome, and ICU admission were all significantly associated with lower odds of fasting the entire Ramadan (OR = 0.25, *p* = 0.019; OR = 0.25, *p* = 0.001; OR = 0.57, *p* = 0.035; and OR = 0.46, *p* = 0.003, respectively). For each new SCD crisis or ER visit, the odds of fasting the entire month decreased by approximately 4.0 and 5.0%, respectively, (OR = 0.96, *p* = 0.017 and OR = 0.95, *p* = 0.003), and having more than four of either was significantly associated with lower likelihood of month-long fasting (OR = 0.45, *p* = 0.001; OR = 0.45, *p* = 0.002, respectively). In addition, older age, male sex, having a blood transfusion history, and having a chronic disease all trended with lower likelihood of month-long fasting; however, none of these parameters achieved significance (*p* > 0.05). No hematological parameters were found to significantly predict fasting the entire Ramadan (*p* > 0.05).

### Impact on health within 30 days after Ramadan

**Figure 2:**
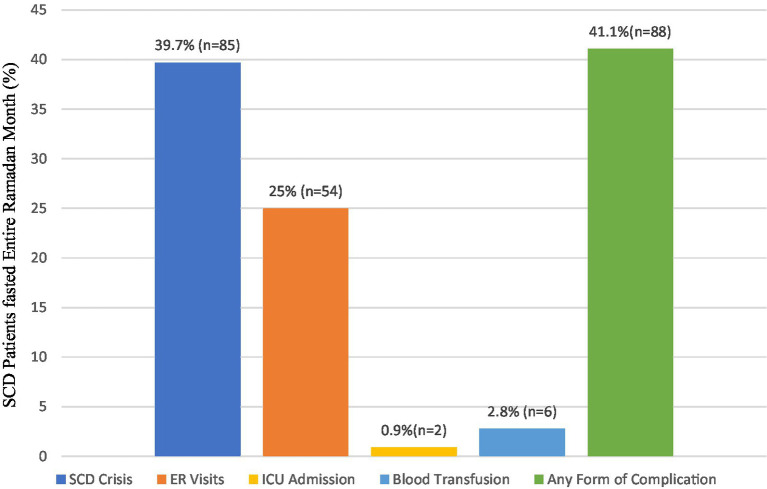
SCD complications within 30 days after Ramadan among SCD patients fasted entire Ramadan month (*n* = 214).

Among patients who fasted for the whole month of Ramadan (*n* = 214), around half (41.1%, *n* = 88) reported experiencing at least one SCD complication within 30 days following the fast. SCD crises accounted for 39.7% of all reported complications, with ER visits coming in second at 25% (Figure 2).

Table 3 presents the regression results concerning predictors of post-Ramadan complications among participants who complete the entire Ramadan fasting (*n* = 214). Participants having secondary and university education exhibited significantly lower likelihood of developing complications (OR = 0.36, *p* = 0.011 and OR = 0.41, *p* = 0.023). Meanwhile, the frequency of SCD crises was a statistically significant predictor of complications (OR = 1.14, *p* < 0.001), with each new crisis increasing the likelihood of complications by approximately 13.6%. Furthermore, participants with more than four SCD episodes had significantly higher odds of complications than those with four or fewer episodes (OR = 3.31, *p* < 0.001). Similarly, participants with a history of emergency visits had more than three times the odds of experiencing complications within 30 days following Ramadan (OR = 3.24, *p* = 0.002), and number of ER visits was a statistically significant predictor, with each additional visit increasing the odds of complications by approximately 8.9% (*p* = 0.005) and participants with more than four ER visits showing significantly higher odds of complications than those with four or fewer visits (OR = 2.55, *p* = 0.004). None of the examined hematological parameters, nor the remaining demographic and health characteristics, significantly predicted the occurrence of post-Ramadan complications (*p* > 0.05).

**Table 3:** Predictors of complications within 30 days after Ramadan among participants with SCD who fasted the entire Ramadan month (*n* = 214).

Variables	*p* value	Odds ratio	95% CI
Age	0.578	1.007	0.98–1.03
Sex (Female)	0.399	1.275	0.72–2.40
Marital (Single)	0.870	0.956	0.55–1.65
Education (Lower than secondary)	0.011	0.356	0.16–0.78
Secondary university	0.023	0.408	0.19–0.88
Chronic diseases	0.451	1.302	0.66–2.58
Number of SCD episodes (*n* = 190)	<0.001	1.136	1.06–1.21
Number of SCD episodes (*n* = 190) Ref. median or less ≤ 4	<0.001	3.312	1.81–6.05
History of emergency visits	0.002	3.243	1.51–6.95
Number of ER Visits (*n* = 167)	0.005	1.089	1.03–1.05
Number of ER Visits (*n* = 167) Ref. median or less ≤ 4	0.004	2.547	1.35–4.78
Previous history of acute chest syndrome	0.406	1.272	0.72–2.24
Previous history of ICU admission	0.413	1.256	0.73–2.17
Previous history of blood transfusion	0.394	1.345	0.68–2.66
Previous history of surgery	0.160	1.517	0.85–2.71
Current use of hydroxyurea	0.349	1.306	0.75–2.28
Optimal dose of hydroxyurea (*n* = 82)	0.502	1.345	0.56–3.22
The use of hydroxyurea during Ramadan	0.469	1.231	0.70–2.16
WBC	0.762	1.010	0.95–1.08
Hemoglobin	0.124	0.855	0.70–1.04
Platelets	0.790	1.000	0.10–1.00
HBA	0.589	0.993	0.97–1.02
HBA2	0.362	1.123	0.87–1.44
HBF	0.273	0.979	0.94–1.02
HBS	0.463	1.009	0.98–1.03

## Discussion

All Muslims globally practice a form of diurnal IF during the month of Ramadan (14). Deciding not to fast during Ramadan or to break your fast mid-day is a serious matter in Islam and should be approached with knowledge, and often consultation. In the current study, majority of the interrupted-fasting group broke their fast based on personal decision, while only a few were medically advised not to complete fasting. These findings highlight a critical need to establish guidelines to assist medical practitioners in providing appropriate fasting recommendations for SCD patients. Furthermore, because many people will fast despite their doctors’ advices, health care professionals and religious/community leaders must collaborate to address these contemporary medical concerns (12).

Despite the fact that inadequate fluid and nutrition intake can trigger SCD symptoms flare-ups, a study from Qatar found that RIF did not impact the frequency of vaso-occlusive or hemolytic crises in these patients (16, 24, 25). Similarly, a case series of four patients practicing different types of IF including complete abstinence from food and/or water, reported no definitive impact on vaso-occlusive pain episodes (aside from one patient who discontinued fasting due to symptom worsening) (26). In line with these findings, not all participants in our study completed the entire month of fasting (averaging 14 h per day). However, our results revealed that RIF had a negative impact on the clinical course of SCD within 30 days following Ramadan. These discrepancies in results regarding the influence of fasting on SCD course might be attributed to various variables, such as the type of fasting used in each study and the variations in participants’ lifestyles. Key lifestyle factors, including hydration, diet, physical activity, rest, and managing stress which can all play significant roles in minimizing symptoms and preventing complications.

SCD presents significant variability among patients, with outcomes ranging from mortality in childhood to the potential for a relatively symptom-free life extending into the eighth decade. The interplay of genetic, environmental, and socioeconomic factors is critical in influencing patient outcomes; however, the precise effects of these variables can be difficult to predict (27, 28). Although there is no correlation between SCD incidence and sex, there have been reports of variations in SCD outcomes among adult patients based on biological sex. Male sex is linked to poorer outcomes in the majority of indicators, such as blood transfusions, hospital admissions, and cerebrovascular complications (29). Furthermore, a recent systematic review by Roky et al. highlighted notable differences in diseases frequency and incidence between both sexes during Ramadan (30). The sex-based difference may account for the female ability to fast for an entire month in the current study. In addition, it might be linked to the fact that women are exempt from fasting during their menstruation, which can last up to 7 days and is equivalent to the average number of interruptions. Moreover, our findings indicate that education level significantly influences safe and effective fasting, and potentially reducing complications. Higher levels of education, particularly secondary school qualifications and above, were more likely to fast the entire month without interruption and to have no post-fasting complications. This result aligns with Shdaifat et al. study, which found that higher education levels are associated with lower management costs for SCD, emphasizing the importance of education in reducing the disease’s overall burden (31). This link is expected, as higher education typically enhances self-management skills, treatment adherence, and symptom control.

Since there is no national or international registry to define or classify the severity of SCD, and lab-based genotyping and phenotyping alone do not reflect clinical severity, assessing the number of complications is essential for evaluating patient risk before fasting and predicting outcomes (32). The current study found that patients with a history of hospitalization or emergency department visits related to SCD crises, particularly those with more than four annual emergency department visits or hospitalization, exhibited higher propensity for interrupted fasting and higher risk of complications within the 30 days following fasting. Individuals with a past incidence of ICU admissions or acute chest syndrome likewise demonstrated decreased likelihood of fasting for the entire month, although the associated risks for post-fasting complications in these groups appear to be negligible.

It is also noteworthy that while hydroxyurea and blood transfusions are well-established therapeutic interventions for SCD patients (28, 33), neither administration of optimal hydroxyurea dose nor history of previous transfusions showed significant correlation with ability to maintain fasting among our patients. This might be because the majority of our patients either not using hydroxyurea or were taking suboptimal doses, preventing optimal treatment benefits.

With regard to hematological parameters, fetal hemoglobin and leukocytosis have been recognized as good indicators of SCD severity and outcomes, but have limited ability in predicting fasting capability (34, 35). A recent study by Ahmed et al. in Qatar demonstrated that fasting results in temporary decreases in platelet and reticulocyte counts, as well as a reversible decline in kidney function during the fasting period. Nevertheless, that study did not succeed in identifying individuals who may be at increased risk for negative outcomes (24). In the current study, only elevated platelet count exhibited close correlation with fasting interruption. This might reflect the baseline thromboinflammatory process in these patients, as thrombocytosis is considered an inflammatory marker with a significant role in some major SCD complications (6, 36).

The results of this study contribute significantly to the literature and highlight that despite the important beneficial impacts of RIF on human health in general, a comprehensive guide is crucial for patients with SCD. Not everyone with SCD should be advised to fast, and decisions should be based on risk categories. The constellation of contributing factors identified in this work could be translated into a scoring system and used to identify a high-risk group and provide proper guidance to the Muslim community suffering from SCD. Furthermore, as fetal hemoglobin level did not show evident impact on fasting success, the findings could be extended to other communities where Arab Indian haplotypes are uncommon (37).

Based on existing literature, this is the first study worldwide to explore the factors influencing RIF among patients with SCD, and the first to assess the health effects of RIF specifically in Saudi SCD patients. Despite that, it has some limitations that should be considered. First, this is a cross-sectional study that can identify associations but cannot prove causality. In addition, participants were from a single center in Saudi Arabia and did not include non-Saudi Muslim patients which may restrict the results’ generalizability. Furthermore, there is the lack of comparison between laboratory markers during and after fasting. Fundamentally, fasting duration is changeable from one country to another and might be affected by seasonal variations, which could also influence guidance. Importantly, the findings of our study apply to adult patients with SCD above the age of 18, while fasting may be undertaken at ages as early as 14 years old. Future studies remain needed for more detailed elucidation of variables and the determination of precise recommendations, as is collaboration between Muslim and non-Muslim countries where SCD is endemic and fasting is a common practice.

In summary, the Ramadan fast serves as a variant of IF that is adopted by adult Muslims globally. The physiological effects of fasting on individuals with SCD are initially subtle but become markedly evident during the post-fasting phase. The ability to predict fasting tolerance and potential complications in the post-fasting period is significantly influenced by a range of sociodemographic factors such as education level as well as clinical variables, including frequency of hospitalizations, emergency department visits, intensive care unit admissions, occurrences of acute chest syndrome, and presence of thrombocytosis. Careful consideration of this clinical profile will enable healthcare providers to offer more informed guidance and optimize fasting decisions for patients.

## Data Availability

The raw data supporting the conclusions of this article will be made available by the authors, without undue reservation.
